# Traffic-Related Air Pollution and Acute Changes in Heart Rate Variability and Respiratory Function in Urban Cyclists

**DOI:** 10.1289/ehp.1003321

**Published:** 2011-06-14

**Authors:** Scott Weichenthal, Ryan Kulka, Aimee Dubeau, Christina Martin, Daniel Wang, Robert Dales

**Affiliations:** 1 Air Health Sciences Division, Health Canada, Ottawa, Canada; 2 Analysis and Air Quality Section, Air Quality Research Division, Environment Canada, Ottawa, Canada; 3 Departments of Medicine and Epidemiology, University of Ottawa, Ottawa, Canada

**Keywords:** black carbon, cycling, heart rate variability, PM_2.5_, traffic pollution, ultrafine particles

## Abstract

Background: Few studies have examined the acute health effects of air pollution exposures experienced while cycling in traffic.

Objectives: We conducted a crossover study to examine the relationship between traffic pollution and acute changes in heart rate variability. We also collected spirometry and exhaled nitric oxide measures.

Methods: Forty-two healthy adults cycled for 1 hr on high- and low-traffic routes as well as indoors. Health measures were collected before cycling and 1–4 hr after the start of cycling. Ultrafine particles (UFPs; ≤ 0.1 μm in aerodynamic diameter), particulate matter ≤ 2.5 μm in aerodynamic diameter (PM_2.5_), black carbon, and volatile organic compounds were measured along each cycling route, and ambient nitrogen dioxide (NO_2_) and ozone (O_3_) levels were recorded from a fixed-site monitor. Mixed-effects models were used to estimate associations between air pollutants and changes in health outcome measures relative to precycling baseline values.

Results: An interquartile range increase in UFP levels (18,200/cm^3^) was associated with a significant decrease in high-frequency power 4 hr after the start of cycling [β = –224 msec^2^; 95% confidence interval (CI), –386 to –63 msec^2^]. Ambient NO_2_ levels were inversely associated with the standard deviation of normal-to-normal (NN) intervals (β = –10 msec; 95% CI, –20 to –0.34 msec) and positively associated with the ratio of low-frequency to high-frequency power (β = 1.4; 95% CI, 0.35 to 2.5) 2 hr after the start of cycling. We also observed significant inverse associations between ambient O_3_ levels and the root mean square of successive differences in adjacent NN intervals 3 hr after the start of cycling.

Conclusions: Short-term exposures to traffic pollution may contribute to altered autonomic modulation of the heart in the hours immediately after cycling.

Cycling is often promoted as a means of reducing traffic congestion and air pollution in urban areas. Indeed, exposure to traffic-related air pollution is known to contribute to adverse respiratory and cardiovascular outcomes, and even modest reductions in air pollution levels resulting from a shift from automobiles to bicycles may have important public health benefits (de Hartog et al. 2010). Nevertheless, individual commuter cyclists may still be exposed to increased levels of air pollution because they are often in close proximity to vehicle emissions. In particular, increased minute ventilation during cycling may result in substantial increases in inhaled doses of fine (≤ 2.5 μm in aerodynamic diameter) particulate matter (PM_2.5_) and ultrafine (≤ 0.1 μm in aerodynamic diameter) particles (UFPs) because vehicle emissions are a major source of these pollutants (Int Panis et al. 2010; Thai et al. 2008; Zuurbier et al. 2010). Furthermore, although few studies have examined the acute health effects of air pollution exposures among cyclists, time spent cycling in traffic was associated with an increased risk of myocardial infarction in a case-crossover study conducted in Germany (Peters et al. 2004).

In the present study we examined the relationship between traffic-related air pollutants and acute changes in heart rate variability (HRV). Decreased HRV has been associated with increased risks of cardiovascular morbidity and mortality (Dekker et al. 1997; Liao et al. 1997; Tsuji et al. 1996), and in general, HRV provides information on the sympathetic- parasympathetic balance of cardiac autonomic modulation (Stein and Kleiger 1999; Task Force 1996). We also examined lung function and exhaled nitric oxide (FE_NO_) and collected on-bicycle personal exposure measures to minimize potential exposure measurement error that may mask the relationship between traffic-related air pollutants and health outcomes (Suh and Zanobetti 2010).

## Materials and Methods

*Participants and study design.* Participants were recruited through advertisements posted across the city of Ottawa, Canada. Eligible participants were healthy nonsmoking men and women who were not exposed to tobacco smoke in the home and did not take medications for preexisting cardiovascular or respiratory conditions.

Study days were scheduled on weekdays between 1030 hours and 1530 hours, and one to two subjects participated on each day. Cycling took place between approximately 1130 hours and 1230 hours, and study days for individual participants were scheduled at least 5 days apart. Participants were asked to take the same route and mode of transportation to and from the study site on each of their three visits. Once at the study site, participants were fitted with a digital Holter monitor and then rested quietly in a sitting position for 30 min. Questionnaires were completed to collect demographic information as well as information on medical history (e.g., allergies, recent medication use), recent illness, alcohol and caffeine consumption, and recent exposure to environmental tobacco smoke. Baseline HRV and respiratory measures were collected immediately after the 30-min rest period. After the rest period, participants were randomly assigned to cycle for 1 hr along a high-traffic route, along a low-traffic route, or indoors. Cycling routes are described in the Supplemental Material (http://dx.doi.org/10.1289/ehp.1003321). Follow-up health measures were collected immediately after cycling and every subsequent hour for 3 hr (i.e., 1–4 hr after the start of cycling). Participants rested quietly in a sitting position for the duration of the postcycling period. The Health Canada Research Ethics Board approved the study protocol, and written informed consent was obtained from each participant.

*Exposure monitoring.* Real-time UFP, PM_2.5_, and black carbon (BC) data were collected using instruments mounted in panniers located on participants’ bicycles. Tubes attached to air intakes of each instrument were run along bicycle frames toward the handlebars in order to collect measurements as close as possible to participants’ breathing zones. Volatile organic compound (VOC) monitors were located on technician’s bicycles traveling immediately in front of participants along each route. One-hour average pollutant concentrations were calculated along each route and were used for the health analyses. In addition, 1-hr average ambient ozone (O_3_), nitrogen dioxide (NO_2_), and sulfur dioxide (SO_2_) data were collected from a fixed monitoring station in downtown Ottawa for the duration of each cycling period (Ontario Ministry of the Environment 2010). A detailed description of exposure monitoring methods is provided in the Supplemental Material (http://dx.doi.org/10.1289/ehp.1003321).

*Heart rate variability.* Electrocardiograms were continuously recorded for the duration of each study day (1030 hours to 1530 hours) using three-channel (seven-lead) digital Holter monitors (Seer Light Extend; GE Medical Systems Information Technologies, Inc., Milwaukee, WI, USA). Data were subsequently analyzed on a GE Medical Systems Information Technology workstation (MARS version 7.2). Time-domain [standard deviation of normal-to-normal (NN) intervals (SDNN), root mean square of successive differences (RMSSD) in adjacent NN intervals, and percentage of adjacent NN intervals differing by > 50 msec (pNN50)] and frequency-domain [low-frequency power (LF; 0.04–0.15 Hz) and high-frequency power (HF; 0.15–0.40 Hz)] measures of HRV were determined for the last 5 min of each segment of the study day: the rest period and 1–4 hr after the start of cycling. HF, RMSSD, and pNN50 reflect parasympathetic modulation of the heart, whereas SDNN reflects total power and LF reflects a mixture of both parasympathetic and sympathetic modulation (Stein and Kleiger 1999; Task Force 1996). The LF:HF ratio is thought to reflect the balance of sympathetic and parasympathetic modulation. Heart rate was also determined for each interval. All of the 5-min segments analyzed contained at least 200 beats, and 92% contained more than 300 beats. Five-minute segments used for HRV analyses occurred before the collection of spirometry and FE_NO_ measures during periods of quiet rest. Beat annotations were automatically assigned by the software and were verified and reviewed by trained technicians at the Arrhythmia Monitoring Center in Ottawa who were blind to exposure status. Beats and intervals were reviewed by trained technicians and only normal sinus beats were used. If the last 5 min of a segment was not suitable for use (< 90% valid recordings), the previous 5-min window was analyzed.

*Respiratory outcomes.* KoKo Legend spirometers (nSpire Health, Longmont, CO, USA) were used to measure forced expiratory volume in 1 sec (FEV_1_), forced vital capacity (FVC), and forced expiratory flow at 25–75% of vital capacity (FEF_25–75_) according to American Thoracic Society (1995) criteria. Niox Mino instruments (Aerocrine, New Providence, NJ, USA) were used to measure FE_NO_; all FE_NO_ measures were collected before spirometry procedures (American Thoracic Society and European Respiratory Society 2005). All respiratory measures were collected by trained technicians, and each subject was assigned to the same instrument and technician on all three visits.

*Statistical analysis.* Linear mixed-effects models were used to examine the relationship between traffic-related air pollutants and changes in health measures 1–4 hr after the start of cycling relative to baseline values measured before cycling. All models included a random intercept for subject to account for correlations between repeated measures collected from individual participants. Separate models were analyzed for each time period, and distributions for baseline changes in health outcomes were approximately normally distributed. Analyses were first conducted using cycling site as the primary exposure variable, with indicator variables used for the high- and low-traffic routes and the indoor site serving as the reference location. Single-pollutant models were then analyzed using continuous measures of mean pollutant levels during each cycling period (UFPs, BC, PM_2.5_, O_3_, NO_2_, and total VOCs). Covariates (age, sex, body mass index, allergies, asthma, ambient temperature during cycling, relative humidity during cycling, day of the week, average heart rate in the hour before HRV measures, average heart rate during cycling, and caffeine or alcohol consumption in the previous 24 hr) were examined one at a time, and only ambient temperature and average heart rate during cycling were retained in final models because these were the only factors that had a meaningful impact on model coefficients (change > 10%). None of the participants reported spending time in a smoking environment at home or elsewhere. Model fit was improved slightly when analyses were conducted using log-transformed data, but the direction of observed associations did not change, and the interpretation of our findings remained consistent. Therefore, nontransformed analyses are presented for ease of interpretation. Models were also examined using percent change in health outcome as the dependent variable, but model fit tended to be better for models using absolute changes, and thus these models are presented.

Indoor data were excluded from models for O_3_ and NO_2_ because indoor measures were not available for these pollutants. In addition, analyses for total VOCs were conducted for all data combined as well as excluding indoor data because total indoor VOCs represent a different mixture than total outdoor VOCs. Analyses were not conducted for SO_2_ or carbon monoxide (CO) because pollutant levels were low and little variation was observed between study days. If an association was detected in single-pollutant models, two-pollutant models were analyzed to examine the stability of model coefficients with the addition of other air pollutants. For O_3_, NO_2_, and total VOCs, two-pollutant models were limited to outdoor data for reasons mentioned above. Separate HRV models were analyzed for UFPs, BC, and PM_2.5_ to include an interaction term for O_3_ because some findings suggest that O_3_ may modify the relationship between PM air pollution and HRV (Fakhri et al. 2009). Centered variables were used to generate interaction terms. Finally, analyses were conducted separately (i.e., stratified analyses) for the high- and low-traffic routes using multivariable linear regression models to evaluate potential confounding by unmeasured route-specific factors. Outcomes and covariates in these models were the same as those in mixed-effects models. Sensitivity analyses were conducted to evaluate the impact of potential outlying values on observed associations, and residual diagnostics were performed to verify model assumptions. All analyses were conducted using STATA (version 11; StataCorp LP, College Station, TX, USA). Model coefficients for specific air pollutants reflect interquartile range (IQR) increases (25th to 75th percentile) in air pollutant levels. An α-value of 0.05 indicated statistical significance.

## Results

Forty-two participants took part in the study over 71 days between May and September 2010. Average ambient temperature and relative humidity values in Ottawa ranged from 16°C to 36°C and from 18% to 80%, respectively, during the study. Participants ranged in age from 19 to 58 years (mean = 35 years) and were predominantly Caucasian (95%), male (67%), and of healthy body weight (mean body mass index, 24.3 kg/m^2^; range, 19.2–41.1 kg/m^2^). Twenty-six participants reported having allergies, and 14 reported asthma diagnosis.

In total, 118 of 126 possible routes were completed throughout the study period, with 38 participants completing all three cycling routes and four participants completing a single route (two low traffic, one high traffic, one indoors). Only 1 hr of Holter monitor data, 2 days of PM_2.5_ data, and 2 days of FE_NO_ data were lost to technician error or malfunctioning equipment during the study. Air pollution exposure data was complete for UFPs, BC, VOCs, and ambient O_3_, NO_2_, and SO_2_ (*n* = 118).

**Figure 1:**
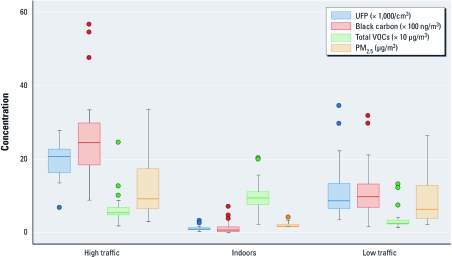
Mean personal air pollution exposures at the high-traffic, low-traffic, and indoor cycling locations. Boxes span the 25th and 75th percentiles, horizontal lines in each box indicate median values, whiskers indicate values within 1.5 IQR of the nearest quartile, and circles indicate values outside this range.

Table 1 summarizes baseline health characteristics for study participants, which are comparable to measures reported in other studies of healthy adults (Chan et al. 2004; Strak et al. 2010; Vallejo et al. 2006). In addition, baseline values were similar before cycling in each separate location [for cycling location-specific baseline values, see Supplemental Material, Table 1 (http://dx.doi.org/10.1289/ehp.1003321)]. On average, mean UFP, BC, and PM_2.5_ concentrations were significantly greater for the high-traffic route than for the low-traffic and indoor locations (Figure 1; for numeric data, see Supplemental Material, Table 2). Average total VOC levels were significantly greater on the high-traffic route than on the low-traffic route, but indoor VOC levels exceeded those for both outdoor cycling locations. Ambient O_3_, NO_2_, and SO_2_ levels were generally low throughout the study period, and levels were not significantly different between high- and low-traffic cycling days (see Supplemental Material, Figure 1). Correlations between air pollutants at each cycling location were low to moderate, with the strongest correlation observed between PM_2.5_ and CO on the high-traffic route (*r* = 0.75; see Supplemental Material, Table 3). A single compound, limonene (a citrus-scented compound used in cleaning agents), was responsible for approximately 25% of total indoor VOC levels (see Supplemental Material, Table 4). As a result, we excluded indoor data from health analyses for total VOCs because total indoor VOCs represented a different mixture than total outdoor VOCs.

**Table 1:** Baseline respiratory and cardiovascular measures.

Table 1. Baseline respiratory and cardiovascular measures.	
Health outcome	Mean ± SD (range)
Respiratory measures	
FE_NO_ (ppb)	22.5 ± 17 (5.5–84.5)
FEV_1_ (L)	3.89 ± 0.83 (2.1–6.0)
FVC (L)	4.91 ± 1.1 (2.5–8.1)
FEF_25–75_ (L)	3.70 ± 0.96 (1.6–6.0)
Cardiovascular measures	
Heart rate (bpm)	71 ± 11 (49–103)
LF (msec^2^)	1,702 ± 1,112 (122–5,700)
HF (msec^2^)	442 ± 513 (11–3,735)
LF:HF	1.31 ± 0.19 (0.97–2.32)
SDNN (msec)	83 ± 33 (26–235)
RMSSD (msec)	37 ± 16 (9–110)
pNN50 (%)	15.5 ± 12 (0–71)
Abbreviations: FE_NO_, exhaled nitric oxide; FEV_1_, forced expiratory volume in 1-second; FVC, forced vital capacity; FEF_25–75,_ forced expiratory flow over the middle half of the FVC; bpm, beats per minute; LF, low frequency; HF, high frequency; SDNN, standard deviation of NN intervals; RMSSD, root mean square of successive differences in adjacent NN intervals; pNN50, proportion of pairs of NN intervals differing by more than 50 msec.	

**Table 2:** Exposure–response slopes for cycling site and changes in HRV from baseline 1–4 hr after the start of cycling relative to indoors.

Table 2. Exposure–response slopes for cycling site and changes in HRV from baseline 1–4 hr after the start of cycling relative to indoors.		
	β-Coefficient (95% CI)	
Outcome	Low traffic	High traffic
ΔLF (msec^2^)		
1 hr	324 (–128 to 775)	427 (–17.1 to 870)
2 hr	306 (–201 to 813)	448 (–54.0 to 950)
3 hr	284 (–221 to 789)	250 (–254 to 753)
4 hr	–364 (–829 to 100)	–316 (–774 to 140)
ΔHF (msec^2^)		
1 hr	3.40 (–174 to 183)	–45.7 (–220 to 129)
2 hr	–67.9 (–248 to 111)	–82.4 (–259 to 94.6)
3 hr	12.7 (–157 to 182)	–36.8 (–203 to 130)
4 hr	–128 (–339 to 81.2)	–225 (–433 to –17.4)*
ΔLF:HF		
1 hr	0.96 (–0.29 to 2.2)	0.67 (–0.56 to 1.9)
2 hr	1.5 (0.11 to 3.0)*	0.83 (–0.56 to 2.2)
3 hr	1.9 (0.40 to 3.3)*	1.3 (–0.16 to 2.7)
4 hr	–1.1 (–2.9 to 0.66)	–0.63 (–2.4 to 1.1)
ΔSDNN (msec)		
1 hr	2.4 (–13 to 18)	3.7 (–11 to 19)
2 hr	4.3 (–10 to 19)	4.9 (–9.6 to 19)
3 hr	10 (–5.6 to 26)	9.5 (–6.4 to 25)
4 hr	5.8 (–8.7 to 20)	11 (–3.7 to 25)
ΔRMSSD (msec)		
1 hr	1.1 (–4.2 to 6.5)	0.36 (–4.9 to 5.6)
2 hr	–1.6 (–6.8 to 3.7)	–1.5 (–6.7 to 3.6)
3 hr	2.9 (–2.4 to 8.3)	1.5 (–3.8 to 6.7)
4 hr	0.098 (–5.2 to 5.4)	–1.4 (–6.6 to 3.8)
ΔpNN50 (%)		
1 hr	0.63 (–3.4 to 4.6)	–0.73 (–4.6 to 3.1)
2 hr	–4.2 (–9.6 to 1.3)	–4.5 (–9.9 to 0.84)
3 hr	2.4 (–1.7 to 6.6)	0.80 (–3.3 to 4.9)
4 hr	0.64 (–3.9 to 5.2)	–1.0 (–5.5 to 3.5)
Separate models were run for each time period, adjusted for ambient temperature and average heart rate during cycling. Model coefficients for the high- and low-traffic sites are in reference to the indoor cycling location. *p < 0.05.		

**Table 3:** Exposure–response slopes*a* for UFPs, BC, and PM_2.5_ levels during cycling and changes in HRV from baseline 1 hr after the start of cycling.

Table 3. Exposure–response slopes*a* for UFPs, BC, and PM_2.5_ levels during cycling and changes in HRV from baseline 1 hr after the start of cycling.						
		β-Coefficient (95% CI)				
Outcome		UFPs		BC		PM_2.5_
ΔLF (msec^2^)						
1 hr		320 (–42 to 684)		172 (–113 to 459)		97 (–200 to 394)
2 hr		319 (–81 to 718)		203 (–112 to 519)		194 (–120 to 509)
3 hr		120 (–286 to 526)		110 (–207 to 428)		200 (–116 to 515)
4 hr		–331 (–700 to 38)		–231 (–520 to 58)		–48 (–342 to 246)
ΔHF (msec^2^)						
1 hr		–110 (–253 to 34)		–28 (–140 to 83)		6.5 (–92 to 105)
2 hr		–140 (–281 to –0.14)*		–37 (–149 to 75)		36 (–73 to 145)
3 hr		–96 (–228 to 37)		–17 (–122 to 85)		18 (–83 to 119)
4 hr		–224 (–386 to –63)*		–94 (–223 to 35)		–44 (–176 to 88)
ΔLF:HF						
1 hr		0.51 (–0.51 to 1.5)		0.38 (–0.40 to –1.2)		0.16 (–0.66 to 0.98)
2 hr		0.94 (–0.23 to 2.1)		0.79 (–0.12 to 1.7)		0.71 (–0.23 to 1.6)
3 hr		1.0 (–0.21 to 2.2)		1.0 (0.11 to 2.0)*		0.43 (–0.55 to 1.4)
4 hr		–0.52 (–2.0 to 0.93)		–0.25 (–1.4 to 0.86)		–0.25 (–1.4 to 0.87)
ΔSDNN (msec)						
1 hr		–0.13 (–13 to 12)		1.9 (–7.8 to 12)		–6.8 (–17 to 2.9)
2 hr		–1.6 (–13 to 9.8)		–1.2 (–10 to 7.8)		–4.3 (–13 to 4.5)
3 hr		–0.85 (14 to 12)		1.7 (–8.3 to 12)		1.2 (–8.8 to 11)
4 hr		3.5 (–8.1 to 15)		4.6 (–4.5 to 14)		–1.5 (–11 to 7.7)
ΔRMSSD (msec)						
1 hr		–1.6 (–5.9 to 2.7)		0.96 (–2.4 to 4.3)		0.73 (–2.6 to 4.1)
2 hr		–2.7 (–6.8 to 1.5)		–0.32 (–3.6 to 2.9)		0.55 (–2.8 to 3.9)
3 hr		–0.85 (–5.1 to 3.4)		0.86 (–2.5 to 4.2)		1.2 (–2.2 to 4.7)
4 hr		–3.3 (–7.5 to 0.95)		–0.19 (–3.5 to 3.1)		–0.55 (–3.9 to 2.8)
ΔpNN50 (%)						
1 hr		–2.2 (–5.4 to 0.98)		–0.055 (–2.5 to 2.4)		0.57 (–2.0 to 3.1)
2 hr		–4.5 (–8.7 to –0.19)*		–2.0 (–5.4 to 1.3)		–1.1 (–4.6 to 2.4)
3 hr		–1.3 (–4.6 to 2.1)		0.32 (–2.3 to 2.9)		0.73 (–1.9 to 3.4)
4 hr		–2.8 (–6.4 to 0.84)		–0.30 (–3.1 to 2.5)		–0.82 (–3.7 to 2.1)
Separate models were run for each time period, adjusted for ambient temperature and average heart rate during cycling. **a**Per IQR: UFPs, 18,200/cm^3^; BC, 1,859 ng/m^3^; PM_2.5_, 8.71 μg/m^3^. *p < 0.05.						

**Table 4:** Exposure–response slopes*a* for O_3_, NO_2_, and total VOC levels during cycling and changes in HRV from baseline 1–4 hr after the start of cycling.

Table 4. Exposure–response slopes*a* for O_3_, NO_2_, and total VOC levels during cycling and changes in HRV from baseline 1–4 hr after the start of cycling.						
		β-Coefficient (95% CI)				
Outcome		O_3_		NO_2_		Total VOCs
ΔLF (msec^2^)						
1 hr		–245 (–603 to 112)		–71 (–383 to 241)		82 (–294 to 28)
2 hr		–81 (–472 to 310)		1.6 (–329 to 332)		173 (–247 to 594)
3 hr		–146 (–548 to 255)		77 (–274 to 429)		–302 (–718 to 115)
4 hr		114 (–267 to 495)		–53 (–471 to 270)		–305 (–700 to 90)
ΔHF (msec^2^)						
1 hr		–144 (–354 to 65)		–56 (–238 to 127)		37 (–183 to 257)
2 hr		–100 (–277 to 78)		–65 (–215 to 85)		40 (–155 to 235)
3 hr		–103 (–259 to 53)		–3.2 (–136 to 130)		8.4 (–160 to 177)
4 hr		–44 (–228 to 140)		–33 (–189 to 123)		27 (–170 to 225)
ΔLF:HF						
1 hr		0.039 (–1.2 to 1.3)		0.76 (–0.28 to 1.8)		–0.59 (–1.9 to 0.69)
2 hr		0.63 (–0.67 to 1.9)		1.4 (0.35 to 2.5)*		0.37 (–0.96 to 1.7)
3 hr		0.55 (–0.90 to 2.0)		1.7 (0.56 to 2.9)*		0.11 (–1.4 to 1.6)
4 hr		0.30 (–1.2 to 1.8)		1.0 (–0.24 to 2.3)		–0.25 (–1.8 to 1.3)
ΔSDNN (msec)						
1 hr		9.0 (–5.1 to 23)		–2.5 (–15 to 9.6)		–0.25 (–1.8 to 1.3)
2 hr		0.068 (–12 to 12)		–10 (–20 to –0.34)*		–2.3 (–15 to 11)
3 hr		–9.0 (–22 to 3.6)		–5.3 (–16 to 5.4)		–6.1 (–20 to 7.6)
4 hr		1.9 (–9.6 to 13)		–1.6 (–11 to 8.2)		1.7 (–11 to 14)
ΔRMSSD (msec)						
1 hr		–4.2 (–9.8 to 1.4)		–1.6 (–6.5 to 3.3)		2.3 (–3.5 to 8.1)
2 hr		–3.7 (–8.3 to 0.88)		–1.6 (–5.5 to 2.3)		0.36 (–4.7 to 5.4)
3 hr		–6.3 (–11 to –1.6)*		0.31 (–4.0 to 4.6)		–1.8 (–7.0 to 3.5)
4 hr		–1.8 (–6.6 to 3.1)		–0.28 (–4.4 to 3.9)		–0.51 (–5.5 to 4.5)
ΔpNN50 (%)						
1 hr		–2.8 (–7.1 to 1.5)		–0.44 (–4.2 to 3.3)		0.15 (–4.3 to 4.6)
2 hr		–3.0 (–6.8 to 0.70)		–1.1 (–4.4 to 2.1)		–0.083 (–4.1 to 4.0)
3 hr		–5.0 (–8.9 to –1.2)*		–0.43 (–3.9 to 3.0)		–1.9 (–6.1 to 2.3)
4 hr		–1.9 (–6.2 to 2.4)		0.17 (–3.5 to 3.9)		–2.3 (–6.7 to 2.2)
Separate models were run for each time period adjusted for ambient temperature and average heart rate during cycling. Models for O_3_, NO_2_, and total VOCs exclude indoor data. **a**Per IQR: O_3_, 15 ppb; NO_2_, 4 ppb; total VOCs, 66 μg/m^3^. *p < 0.05.						

*Heart rate variability.* Analysis by cycling site. HF power was decreased after cycling on the high-traffic route relative to indoors, with a significant decrease observed at the 4-hr time point (Table 2). LF:HF was significantly increased 2 and 3 hr after the start of cycling on the low-traffic route relative to indoors but not on the high-traffic route (Table 2). We identified one influential data point in the analysis of HF, as one participant experienced a large decrease at the 4-hr time point after cycling on the high-traffic route. When we removed this value, the direction of the observed association with high-traffic versus indoor cycling remained consistent but the magnitude decreased and was no longer statistically significant [β = –141; 95% confidence interval (CI), –302 to 20.6]. Cycling site was not associated with significant changes in other measures of HRV.

*Analysis by specific air pollutants.* We observed consistent inverse relationships between an IQR increase in UFPs and HF, with significant decreases observed at the 2- and 4-hr time points (Table 3). UFPs were also inversely associated with pNN50, with a significant decrease observed 2 hr after the start of cycling (Table 3). We observed nonstatistically significant inverse associations between BC and HF, but these relationships disappeared when we included UFPs with BC in two-pollutant models, whereas coefficients for UFPs remained stable (data not shown). We identified one outlying data point in the analysis of UFPs and HF at the 2-hr time point, and when we removed this point the observed association was no longer statistically significant (β = –86.0 msec^2^; 95% CI, –201 to 28.9 msec^2^). We also identified one outlying data point in the analysis of UFPs and HF at the 4-hr time point, but the observed association remained statistically significant when we removed this point (β = –183 msec^2^; 95% CI, –307 to –59.4 msec^2^). Further adjustment for other air pollutants in two-pollutant models did not change the observed association between UFPs and HF or pNN50.

IQR increases in ambient NO_2_ levels were associated with significantly increased LF:HF at the 2- and 3-hr time points (Table 4), and these relationships remained stable when adjusted for other air pollutants (data not shown). BC was also associated with a significant increase in LF:HF 3 hr after the start of cycling (Table 3), but this relationship decreased and was no longer statistically significant when we included NO_2_ in the model (data not shown). Ambient NO_2_ levels were associated with decreased SDNN 1–4 hr after the start of cycling, with a significant decrease observed at the 2-hr time point (Table 4). This relationship remained stable when we included other air pollutants in the model.

We observed significant inverse relationships between O_3_ and RMSSD and pNN50 at the 3-hr time point (Table 4). Further adjustment for other air pollutants did not change these associations, and O_3_ did not appear to modify effects of UFPs, BC, or PM_2.5_ in the analysis of HRV (*p* > 0.05 for interaction terms; data not shown). Total VOC exposures during cycling were not associated with HRV (Table 4). In general, the observed associations remained consistent when we conducted the above analyses separately for the high- and low-traffic routes (data not shown).

*Respiratory outcomes.* We did not observe strong relationships between traffic-related air pollution and acute changes in respiratory outcomes [see Supplemental Material, Tables 5–7 (http://dx.doi.org/10.1289/ehp.1003321)]. PM_2.5_ was associated with a small increase in FE_NO_ 2 hr after cycling (β = 1.1 ppb; 95% CI, 0.08–2.2 ppb), and UFPs were associated with increased FEF_25–75_ at the 1-hr time point (β = 191 mL; 95% CI, 10–371 mL). BC, O_3_, and total VOCs were not associated with significant changes in respiratory outcomes, but NO_2_ levels were associated with significantly increased FEV_1_ 2 hr (β = 121 mL; 95% CI, 27–216 mL) and 3 hr (β = 129 mL; 95% CI, 23–234 mL) after the start of cycling.

## Discussion

Our findings suggest that short-term exposure to traffic-related air pollution may contribute to changes in the autonomic regulation of the heart in the hours immediately after cycling. Specifically, UFP exposures were inversely associated with HF and pNN50, O_3_ was inversely associated with RMSSD and pNN50, and NO_2_ was inversely associated with SDNN and positively associated with LF:HF. In general, these findings suggest decreased parasympathetic modulation of the heart in response to traffic-related air pollution. Although some findings may have occurred by chance owing to multiple comparisons, consistency in the direction (i.e., reduced parasympathetic modulation) of observed associations for multiple HRV measures tends to suggest a true association. As in previous panel studies of healthy cyclists (Jacobs et al. 2010; Strak et al. 2010), we did not observe strong associations between traffic-related air pollution and acute changes in respiratory outcomes.

We did not identify other studies examining the relationship between HRV and air pollution exposures among cyclists; however, 1- to 4-hr moving averages of personal UFP exposures were associated with decreased HF, LF, RMSSD, and SDNN in a panel of young adults and elderly subjects in Taiwan (Chan et al. 2004). Although our findings for UFPs and HF are consistent with these results, we did not observe significant associations between UFPs and LF, RMSSD, or SDNN; however, we observed consistent inverse relationships for RMSSD. One explanation for this discrepancy may be differences in PM composition, because this study specifically targeted exposures in traffic, whereas Chan et al. (2004) monitored exposures throughout normal daily activities. As in this study, Timonen et al. (2006) reported associations between NO_2_ and LF:HF, but they did not observe a significant inverse relationship between UFPs and HF. Likewise, other studies have also failed to detect significant relationships between UFPs and HRV (Park et al. 2005; Schneider et al. 2010); however, non-statistically significant inverse relationships between UFPs and HF were reported in both of these studies. One explanation for this discrepancy may be increased exposure measurement error owing to the use of fixed-site UFP monitors in some of these studies (Park et al. 2005; Schneider et al. 2010; Suh and Zanobetti 2010). Alternatively, differences in population characteristics (i.e., healthy subjects vs. elderly subjects or those with cardiovascular disease) and UFP exposure averaging times may also contribute to discrepancies between studies.

A number of studies have reported inverse associations between HRV and ambient PM_2.5_, BC (or elemental carbon), organic carbon (OC), NO_2_, and O_3_, particularly among elderly subjects and those with preexisting cardiovascular disease (Adar et al. 2007; Chuang et al. 2007; Creason et al. 2001; Gold et al. 2000; Liao et al. 1999; Luttmann-Gibson et al. 2006; Magari et al. 2002; Park et al. 2005, 2010; Pope et al. 2004; Schneider et al. 2010; Schwartz et al. 2005; Timonen et al. 2006; Vallejo et al. 2006; Zanobetti et al. 2010). Although our findings for NO_2_ and O_3_ are generally consistent with previous evidence suggesting decreased HRV with increased exposure (Chan et al. 2005; Gold et al. 2000; Liao et al. 2004; Park et al. 2005; Timonen et al. 2006), we did not observe a significant relationship between total VOCs (organic carbon) and HRV as previously reported (Chuang et al. 2007; Schneider et al. 2010). Specifically, recent studies have observed significant inverse associations between OC and HF, RMSSD, and pNN50 (Schneider et al. 2010) and between OC and SDNN, RMSSD, LF, and HF (Chuang et al. 2007). However, both of these studies were conducted among patients with preexisting cardiovascular disease, so disease status may be an important effect modifier in the relationship between OC and HRV.

We did not observe significant associations between BC or PM_2.5_ and HRV. This is consistent with previous findings from a controlled crossover study of diesel exhaust exposure (Peretz et al. 2008) but is in contrast to previous studies of traffic-related air pollution (Riediker et al. 2004; Wu et al. 2010; Zanobetti et al. 2010). However, time spent in traffic 2 hr before HRV measures was the strongest predictor of decreased HF and RMSSD in one of these studies (Zanobetti et al. 2010), and our findings for HF and high-traffic cycling generally support this result. Other studies have also reported decreased HRV in response to short-term traffic-related exposures (Adar et al. 2007); thus, it seems plausible that decreased HRV may play a role in previously reported associations between short-term traffic exposures and onset of myocardial infarction (Peters et al. 2004).

Although the use of personal exposure measures, real-life cycling conditions, and a crossover design were important strengths of this investigation, it is important to recognize several limitations. First, we could not control for personal exposures experienced en route to the study site, so it is possible that these exposures might have influenced the results. To address this concern, we asked participants to take the same route and mode of transportation to the site each day. In addition, the use of changes from baseline in health measures as the primary outcome likely minimized the impact of precycling exposures because outcomes measured at hourly intervals throughout the day were likely affected similarly by exposures en route to the study site. Second, we could not adjust for the effects of respiration on HRV, a factor that is particularly important for HF because this measure is sensitive to respiratory variation (Task Force 1996). However, we controlled study conditions as much as possible and asked participants to rest quietly in a sitting position for the duration of pre- and postcycling periods. Although we cannot rule out potential confounding by unmeasured respiration effects, such effects would have to be correlated with air pollution levels along cycling routes in order to confound the results. Third, our findings are based on a relatively small sample of subjects, and as a result, measures of association were often imprecise and may not be generalizable to the broader population. In addition, we had limited power to detect potentially important interactions between air pollutants such as those previously reported between PM air pollution and O_3_ (Fakhri et al. 2009). Fourth, we did not examine the impact of traffic-related air pollution exposures beyond 4 hr after the start of cycling, and we did not have data on other cardiovascular measures such as ST-segment depression (indicative of cardiac ischemia) and deceleration capacity that have recently been associated with air pollution (Delfino et al. 2011; Schneider et al. 2010). Because we detected significant relationships 4 hr after the start of cycling, it seems plausible that the observed effects might have continued over longer time periods, and future studies should evaluate this possibility. Finally, personal exposure measures were not available for NO_2_ and O_3_, and we assigned each subject who participated on the same study day the same ambient concentration; therefore, exposure measurement error likely resulted in underestimation of effect estimates for these pollutants.

To our knowledge, this is the first study to examine the relationship between traffic-related air pollution exposures and acute changes in HRV among cyclists. Although a number of studies have observed inverse associations between ambient air pollution and HRV in elderly populations and/or subjects with preexisting cardiovascular disease, our findings suggest that short-term exposure to traffic may have a significant impact on cardiac autonomic function in healthy adults. The clinical implications of this relationship are unclear because the health benefits of cycling might outweigh any potential negative impact that acute reductions in HRV may have in healthy adults (de Hartog et al. 2010). Indeed, altered HRV may be a single component of a more complex causal pathway that requires a series of other cofactors such as endothelial dysfunction and individual susceptibilities (i.e., genetic predisposition, health status) to ultimately result in cardiovascular morbidities associated with air pollution (Brook 2008, 2010). However, our findings suggest that, when possible, it may be prudent to select cycling routes that reduce exposure to traffic and to avoid cycling outdoors or to exercise indoors on days with elevated air pollution levels. In addition, the planning of new cycling routes/bicycle paths in urban areas should aim to minimize time spent in high-traffic areas in order to reduce exposures of recreational riders who may be more susceptible (e.g., elderly) to the acute cardiovascular health effects of traffic-related air pollution.

## Supplemental Material

(172 KB) PDF
