# Supplementary material for: Traffic-Related Air Pollution and Acute Changes in Heart Rate Variability and Respiratory Function in Urban Cyclists
Source: Environ Health Perspect. 2011 Jun 14;119(10):1373–8. doi: 10.1289/ehp.1003321 (PMC3230442; doi:10.1289/ehp.1003321)

Supplemental Material

Traffic-Related Air Pollution and Acute Changes in Heart Rate Variability and Respiratory Function in Urban Cyclists

Scott Weichenthal[1]*, Ryan Kulka[1], Aimee Dubeau[1], Christina Martin[1], Daniel Wang[2], Robert Dales[3]

[1]Health Canada, Air Health Sciences Division, Ottawa, Canada

[2] Environment Canada, Air Quality Research Division, Analysis and Air Quality Section, Ottawa, Canada

[3] University of Ottawa Departments of Medicine and Epidemiology, Ottawa, Canada

*Corresponding Author

Dr. Scott Weichenthal

Health Canada Air Health Sciences Division

269 Laurier Avenue West, AL 4903C

Ottawa, ON

K1A 0K9

Telephone: (613) 948-7765

Fax: (613) 948-8482

Email: scott.weichenthal@hc-sc.gc.ca

Table of Contents

Supplemental Materials and Methods 3

Supplemental Material, Table 1. Baseline respiratory and cardiovascular measures by cycling site (Mean ± SD (Range)) 6

Supplemental Material, Table 2. Air pollutant levels at each cycling location 7

Supplemental Material, Table 3: Spearman's correlations for air pollutants monitored at each site 8

Supplemental Material, Table 4: Twenty most common individual VOCs at each site 9

Supplemental Material, Table 5: Mixed-effect model slopes for the relationship between cycling site and baseline changes in acute respiratory outcomes 1 to 4-hours after the start of cycling 10

Supplemental Material, Table 6. Mixed-effect model slopes[a] for UFPs, BC, and $PM_{2.5}$ and baseline changes in acute respiratory outcomes 1 to 4-hours after the start of cycling 11

Supplemental Material, Table 7. Mixed-effect model slopes[a] for ambient O_3 and NO_2 levels during cycling and baseline changes in acute respiratory outcomes 1 to 4-hours after the start of cycling 12

Supplemental Material, Figure 1. Ambient ozone, nitrogen dioxide, and sulfur dioxide levels on high and low-traffic cycling days. 13

Supplemental Materials and Methods

Cycling Routes

High and low-traffic routes were approximately 10 km in length and participants were guided along each route by a study technician. When possible, technicians maintained a pace of approximately 20 km/h in order to complete each route in approximately 1-hour. Outdoor cycling was cancelled on rainy days. The high-traffic route covered a portion of the downtown core of Ottawa including roadways with dedicated lanes for public transit buses. The low-traffic route was a bicycle path along the Ottawa River away from traffic with the exception of a short section of roadway (< 1 km) between the central study site and the bicycle path. The indoor site was located in an empty boardroom of the main study building and a stationary cycling apparatus was used to mount outdoor bicycles for indoor use. A bicycle-mounted video camera recorded each participant's high-traffic route and participants watched this video to mimic outdoor cycling activities during indoor cycling (i.e. starting and stopping).

Exposure Monitoring

Air pollution exposure monitoring was conducted using instruments mounted in panniers located on participants' bicycles. Tubes attached to air intakes of each instrument were run along bicycle frames towards the handle bars in order to collect measurements as close as possible to participants' breathing zones. Participants used the same instrument group for each cycling session.

Particulate Air Pollution

Ultrafine particle (0.01-1 µm) number concentration data were collected using TSI Model 3007 condensation particle counters (TSI, St Paul, MN, USA). Tilt sensors on these instruments were disabled according to instructions provided by the manufacturer.

This modification was necessary as these instruments sometimes mistake occasional bumps during cycling as “tilt” causing the instrument to stop logging data. This modification involved soldering a single wire to bypass the tilt sensor. In side-by-side comparisons before and after the study, instruments with the tilt sensor disabled provided the same readings as those without the modification (data not shown). $PM_{2.5}$ data were collected using TSI Dust Trak monitors (TSI, St Paul, MN, USA). Dust Trak monitors and TSI Model 3007 instruments were zero-checked daily and inlets were cleaned weekly. Real-time UFP and $PM_{2.5}$ data were logged at 1-second sampling intervals and $PM_{2.5}$ data were corrected for overestimation by the Dust Trak relative to gravimetric methods (Wallace et al. 2010). Black carbon (BC) data were collected at 1-minute sampling intervals using MicroAeth Model AE51 aethalometers equipped with high volume sampling pumps (800 mL/min) (Magee Scientific, Berkeley, CA, USA). One-hour averages were calculated from 1-second and 1-minute sampling intervals and these averages were used in the health analysis.

Volatile Organic Compounds (VOCs)

VOC data were collected along each route using 1-L SUMMA canisters placed in panniers located on technicians’ bicycles travelling directly in front of participants. VOC canisters were placed near participants during indoor cycling. VOC analysis was conducted by GC-MS according to the United States Environmental Protection Agency method TO-15. Total VOC levels along each route were calculated as the sum of individual VOCs in each sample; method detection limits ranged from 0.01 $\mu g/m^3$ to 0.09 $\mu g/m^3$ for individual VOCs. Exposure and health analyses for individual VOCs will be reported separately.

Other Exposure Measures

Carbon monoxide was measured using the Langan Enhanced CO Measurer Model T15n (Langan Products, San Francisco, CA, USA) and temperature and relative humidity data were collected using HOBO Data Loggers (Onset, Cape Cod, MA, USA). Carbon monoxide, temperature, and relative humidity were sampled at 1-minute logging intervals. Finally, 1-hour average ambient O_3, NO_2, and SO_2 data were collected from a fixed monitoring station in downtown Ottawa (approximately 2 km from the main study site) for the duration of each cycling period. If two subjects participated on the same day both were assigned the same ambient concentration of O_3, NO_2, and SO_2. Consistent readings were observed between all monitors of the same type in side-by-side measurements collected prior to starting the study (data not shown).

Supplemental Tables

Supplemental Material, Table 1. Baseline respiratory and cardiovascular measures by cycling site (Mean ± SD (Range))

Health Outcome	Cycling Location		
	High Traffic Route	Low Traffic Route	Indoors
Respiratory Measures			
FE_{NO} (ppb)	22.1 ± 16 (6.5-74)	22.9 ± 18 (5.5-83)	22.4 ± 18 (5.5-84.5)
FEV_1 (L)	3.85 ± 0.82 (2.1-5.6)	3.92 ± 0.85 (2.2-6.0)	3.91 ± 0.83 (2.2-5.8)
FVC (L)	4.90 ± 1.0 (2.5-7.7)	4.89 ± 1.1 (2.6-8.1)	4.92 ± 1.1 (2.6-7.5)
FEF_{25-75} (L)	3.60 ± 0.91 (1.8-5.4)	3.80 ± 0.99 (2.0-6.0)	3.68 ± 1.0 (1.6-5.7)
Cardiovascular Measures			
Heart Rate (bpm)	71 ± 11 (49-100)	72 ± 12 (49-100)	70 ± 11 (52-103)
LF (ms^2)	1698 ± 1091 (122-5700)	1529 ± 918 (267-5272)	1882 ± 1298 (254-4988)
HF (ms^2)	474 ± 651 (11-3735)	406 ± 443 (22-2313)	446 ± 425 (12-2139)
LF/HF (none)	5.8 ± 3.8 (0.81-16)	6.0 ± 3.9 (1.0-18)	5.9 ± 4.5 (1.2-27)
SDNN (ms)	82 ± 36 (26-235)	77 ± 24 (37-154)	91 ±38 (29-196)
RMSSD (ms)	38 ± 17 (9-110)	35.5 ± 15 (11-76)	39 ± 16 (10-89)
pNN50 (%)	16 ± 13 (0-71)	14.2 ± 12 (0-43.9)	17 ± 12 (0-54)

Abbreviations: FE_{NO}, exhaled nitric oxide; FEV_1, forced expiratory volume in 1-second; FVC, forced vital capacity; FEF_{25-75}, forced expiratory flow over the middle half of the FVC; bpm, beats per minute; LF, low frequency; HF, high frequency; SDNN, standard deviation of NN intervals; RMSSD, root mean square of successive differences in adjacent NN intervals; pNN50, proportion of pairs of NN intervals differing by more than 50 ms.

Supplemental Material, Table 2. Air pollutant levels at each cycling location

Air Pollutant	Cycling Location				
	High Traffic Route		Low Traffic Route		Indoors
	Mean (Range)	Mean Differences (95% CI)	Mean (Range)	Mean Difference (95% CI)	Mean (Range)
UFPs (no./cm^3)	19,747 (6834-27,800)	[a] 8864 (6229, 11,498)<br>[b] 18,584 (17,186, 19,982)	10,882 (3590-34,000)	[b] 9720 (7428, 12,011)	1162 (413-3210)
BC (ηg/m^3)	2520 (890-5670)	[a] 1440 (1051, 1829)<br>[b] 2382 (2042, 2721)	1079 (173-3197)	[b] 941 (727, 1156)	138 (1-720)
$PM_{2.5}$ ($\mu g/m^3$)	12.2 (3.0-34)	[a] 4.12 (1.1, 7.1)<br>[b] 10.3 (7.8, 12.7)	8.14 (2.2-26)	[b] 6.15 (4.3, 8.0)	2.00 (1.5-4.1)
CO (ppm)	1.4 (0.6-2.6)	[a] 0.42 (0.24, 0.60)<br>[b] 0.29 (0.12, 0.46)	0.915 (0.5-1.5)	[b] -0.13 (-0.24, -0.02)	1.08 (0.78-1.6)
Ambient O_3 (ppb)	34 (13-52)	[a] 2.0 (-2.2, 6.2)<br>[b] 6.6 (2.3, 10.9)	32 (3-51)	[b] 4.6 (0.36, 8.8)	27 (9-52)
Ambient NO_2 (ppb)	4.8 (1-11)	[a] 0.19 (-0.91, 1.3)<br>[b] 0.03 (-1.2, 1.3)	4.6 (1-10)	[b] -0.17 (-1.4, 1.0)	4.8 (1-11)
Ambient SO_2 (ppb)	1.0 (0-2)	[a] -0.23 (-0.5, 0.05)<br>[b] -0.15 (-0.4, 0.1)	1.2 (0-4)	[b] 0.07 (-0.2, 0.4)	1.1 (0-2)
Total VOCs ($\mu g/m^3$)	63.8 (18-246)	[a] 30.4 (16, 44)<br>[b] -34.9 (-53, -16)	33.4 (15-132)	[b] -65.3 (-82,-48)	98.7 (22-204)

[a] Relative to the low-traffic route; [b] Relative to indoors

Supplemental Material, Table 3: Spearman's correlations for air pollutants monitored at each site

	UFP	$PM_{2.5}$	BC	CO	SO_2[a]	NO_2[a]	O_3[a]
High Traffic							
Total VOCs	-0.41	-0.50	-0.40	0.19	-0.30	0.12	0.13
UFP		0.06	0.37	0.09	0.01	0.62	0.02
$PM_{2.5}$			0.32	0.75	-0.30	0.31	0.58
BC				0.29	-0.19	0.47	-0.04
CO					-0.60	0.31	0.54
SO_2[a]						0.06	-0.33
NO_2[a]							0.17
Low Traffic							
Total VOCs	-0.36	-0.25	-0.43	0.15	0.08	0.35	-0.06
UFP		-0.22	0.42	0.0	0.03	0.51	0.22
$PM_{2.5}$			0.24	0.59	-0.04	0.45	0.36
BC				-0.04	0.28	0.48	0.19
CO					-0.34	0.32	0.39
SO_2[a]						-0.13	-0.13
NO_2[a]							0.32
Indoors							
Total VOCs	0.19	0.44	0.60	0.50	-0.29	0.55	0.09
UFP		0.38	0.52	-0.06	0.29	0.24	0.08
$PM_{2.5}$			0.18	0.25	0.16	0.24	0.42
BC				-0.18	0.114	0.32	0.34
CO					-0.28	-0.08	0.17
SO_2[a]						-0.04	0.01
NO_2[a]							-0.02

[a] Ambient measures from a fixed site monitor in downtown Ottawa.

Supplemental Material, Table 4: Twenty most common individual VOCs at each site

High Traffic		Low-Traffic		Indoors	
Compound	Median (μg/m^3)	Compound	Median (μg/m^3)	Compound	Median (μg/m^3)
2-Methylbutane	4.21	Freon 12	2.55	Limonene	23.16
Toluene	3.40	Ethane	1.90	Isoprene	4.69
Propane	2.94	Freon 11	1.53	2-Methylbutane	3.85
Ethane	2.86	Propane	1.26	1,4-Dichlorobenzene	3.53
Freon 12	2.62	Chloromethane	1.19	Toluene	3.50
Butane	2.59	2-Methylbutane	1.15	Freon22	2.65
Ethylene	2.58	Toluene	1.07	Pentane	2.65
Freon 11	1.53	Freon 22	1.06	Ethane	2.60
m,p-Xylene	1.37	Isoprene	0.86	Freon 12	2.55
Freon 22	1.36	Ethylene	0.81	Propane	2.46
Isobutane	1.28	Butane	0.80	m, p-Xylene	1.79
Chloromethane	1.21	Freon 113	0.66	Isobutane	1.77
2-Methylpentane	1.02	Pentane	0.56	Ethylene	1.56
Benzene	0.94	Limonene	0.51	Freon 11	1.54
Propene	0.90	Carbon tetrachloride	0.47	Chloromethane	1.39
Hexane	0.81	Isobutane	0.45	3-Methylhexane	1.07
3-Methylpentane	0.77	t-2-Octene	0.42	Trichloroethene	1.04
1-Butene	0.76	Benzene	0.34	2-Methylpentane	0.94
Limonene	0.72	1-Butene	0.31	Hexane	0.92
Freon 113	0.65	Dichloromethane	0.31	o-Xylene	0.84

Freon 11, Trichlorofluoromethane; Freon 12, Dichlorodifluoromethane; Freon 22, Chlorodifluoromethane; Freon 113, 1,1,2-Trichlorotrifluoroethane

Supplemental Material, Table 5: Exposure response slopes for cycling site and changes in respiratory outcomes from baseline 1 to 4-hours after the start of cycling relative to indoors

Outcome	Time	Low Traffic β (95% CI)	High Traffic β (95% CI)
ΔFE_{NO} (ppb)	1-hour	-1.9 (-3.7, -0.11)*	-0.65 (-2.4, 1.1)
	2-hours	-0.13 (-1.8, 1.5)	1.4 (-0.20, 3.0)
	3-hours	0.70 (-0.76, 2.1)	0.49 (-0.95, 1.9)
	4-hours	0.34 (-1.4, 2.1)	0.68 (-1.1, 2.4)
ΔFEV_1 (mL)	1-hour	52 (-66, 169)	48 (-68, 163)
	2-hours	-8.7 (-134, 117)	74 (-50, 198)
	3-hours	-4.8 (-141, 131)	64 (-70, 198)
	4-hours	36 (-58, 131)	79 (-15, 172)
ΔFVC (mL)	1-hour	13 (-132, 159)	108 (-36, 252)
	2-hours	157 (-48, 363)	137 (-66, 34)
	3-hours	-0.41 (-125, 124)	30 (-94, 153)
	4-hours	5.6 (-121, 132)	29 (-96, 155)
ΔFEF_{25-75} (mL)	1-hour	104 (-129, 337)	141 (-89, 371)
	2-hours	-45 (-324, 235)	63 (-214, 340)
	3-hours	86 (-181, 354)	69 (-195, 333)
	4-hours	109 (-112, 331)	83 (-136, 302)

Separate models were run for each time period adjusted for ambient temperature and average heart rate during cycling. Model coefficients for the high and low traffic sites are in reference to the indoor cycling location. *Statistically significant ($p<0.05$).

Supplemental Material, Table 6. Exposure-response slopes[a] for UFPs, BC, and $PM_{2.5}$ levels during cycling and changes in respiratory outcomes from baseline 1 to 4-hours after the start of cycling

Outcome	Time	UFP β (95% CI)	BC β (95% CI)	$PM_{2.5}$ β (95% CI)
ΔFE_{NO} (ppb)	1-hour	-0.30 (-1.8, 1.2)	-0.33 (-1.5, 0.80)	0.37 (-0.81, 1.5)
	2-hours	0.71 (-0.62, 2.0)	0.78 (-0.24, 1.8)	1.1 (0.080, 2.2)*
	3-hours	0.067 (-1.1, 1.2)	-0.37 (-1.3, 0.53)	0.38 (-0.56, 1.3)
	4-hours	0.089 (-1.3, 1.5)	-0.30 (-1.4, 0.77)	0.38 (-0.70, 1.5)
ΔFEV_1 (mL)	1-hour	71 (-22, 164)	17 (-56, 90)	-16 (-90, 58)
	2-hours	41 (-58,140)	21 (-58, 100)	32 (-46, 110)
	3-hours	87 (-20, 193)	27 (-57, 111)	4.9 (-81, 90)
	4-hours	69 (-4.7, 143)	31 (-28, 90)	10 (-50, 69)
ΔFVC (mL)	1-hour	69 (-45, 184)	53 (-37, 143)	-23 (-170, 124)
	2-hours	93 (-73, 260)	-28 (-100, 157)	46 (-84, 175)
	3-hours	35 (-63, 133)	2.2 (-75, 79)	-17 (-90, 56)
	4-hours	-3.6 (-104, 96)	30 (-75, 81)	2.5 (-75, 79)
ΔFEF_{25-75} (mL)	1-hour	191 (10, 371)*	56 (-89, 200)	-23 (-170, 124)
	2-hours	61 (-152, 275)	-1.6 (-169, 166)	-36 (-205, 132)
	3-hours	108 (-103, 318)	-4.5 (-174, 165)	-2.7 (-169, 163)
	4-hours	125 (-48, 298)	-16 (-153, 121)	-62 (-204, 80)

Separate models were run for each pollutant and time period adjusted for ambient temperature and average heart rate during cycling.[a] Per IQR: UFPs: 18,200 particles/cm^3; BC: 1859 ng/m^3; $PM_{2.5}$: 8.71 $\mu g/m^3$. *Statistically significant ($p<0.05$).

Supplemental Material, Table 7. Exposure-response slopes[a] for O_3, NO_2, and Total VOC levels during cycling and changes in respiratory outcomes from baseline 1 to 4-hours after the start of cycling

Outcome	Time	O_3 β (95% CI)	NO_2 β (95% CI)	Total VOCs β (95% CI)
ΔFE_{NO} (ppb)	1-hour	0.67 (-1.2, 2.6)	-0.16 (-1.7, 1.4)	1.1 (-0.90, 3.0)
	2-hours	0.83 (-0.90, 2.6)	0.059 (-1.4, 1.5)	1.3 (-0.50, 3.0)
	3-hours	0.49 (-0.96, 1.9)	-0.23 (-1.4, 0.96)	-0.87 (-2.4, 0.63)
	4-hours	0.28 (-1.2, 1.8)	-0.95 (-2.1, 0.24)	-0.087 (-1.6, 1.4)
ΔFEV_1 (mL)	1-hour	6.8 (-103, 117)	72 (-20, 165)	-49 (-163, 64)
	2-hours	11 (-105, 127)	121 (27, 216)*	26 (-99, 152)
	3-hours	-81 (-209, 47)	129 (23, 234)*	-5.0 (-145, 135)
	4-hours	-67 (-149, 15)	53 (-16, 123)	-10 (-100, 79)
ΔFVC (mL)	1-hour	16 (-94, 126)	17 (-77, 111)	27 (-91, 146)
	2-hours	-52 (-245, 141)	16 (-146, 180)	-14 (-226, 197)
	3-hours	43 (-53, 138)	45 (-35, 125)	0.39 (-103, 104)
	4-hours	22 (-81, 126)	63 (-24, 150)	-7.0 (-119, 105)
ΔFEF_{25-75} (mL)	1-hour	7.4 (-202, 217)	0.42 (-176, 176)	51 (-177, 279)
	2-hours	-15 (-242, 212)	-7.9 (-200, 184)	0.60 (-244, 245)
	3-hours	-118 (-157, 85)	108 (-63, 280)	-76 (-297, 145)
	4-hours	-30 (-209, 148)	-92 (-241, 58)	-110 (-301, 82)

Separate models were run for each time period adjusted for ambient temperature and average heart rate during cycling. Models for O_3, NO_2, and Total VOCs exclude indoor data. [a] Per IQR: O_3: 15 ppb ; NO_2: 4 ppb; Total VOCs: 66 ug/m^3. *Statistically significant ($p<0.05$).

Supplemental Figures

Supplemental Material, Figure 1. Ambient ozone, nitrogen dioxide, and sulfur dioxide levels on high and low-traffic cycling days. The shaded box spans the 25th and 75th percentiles with horizontal lines in each box indicating median values. Upper and lower adjacent lines indicate values within 1.5 IQR of the nearest quartile. Dots indicate values outside this range.

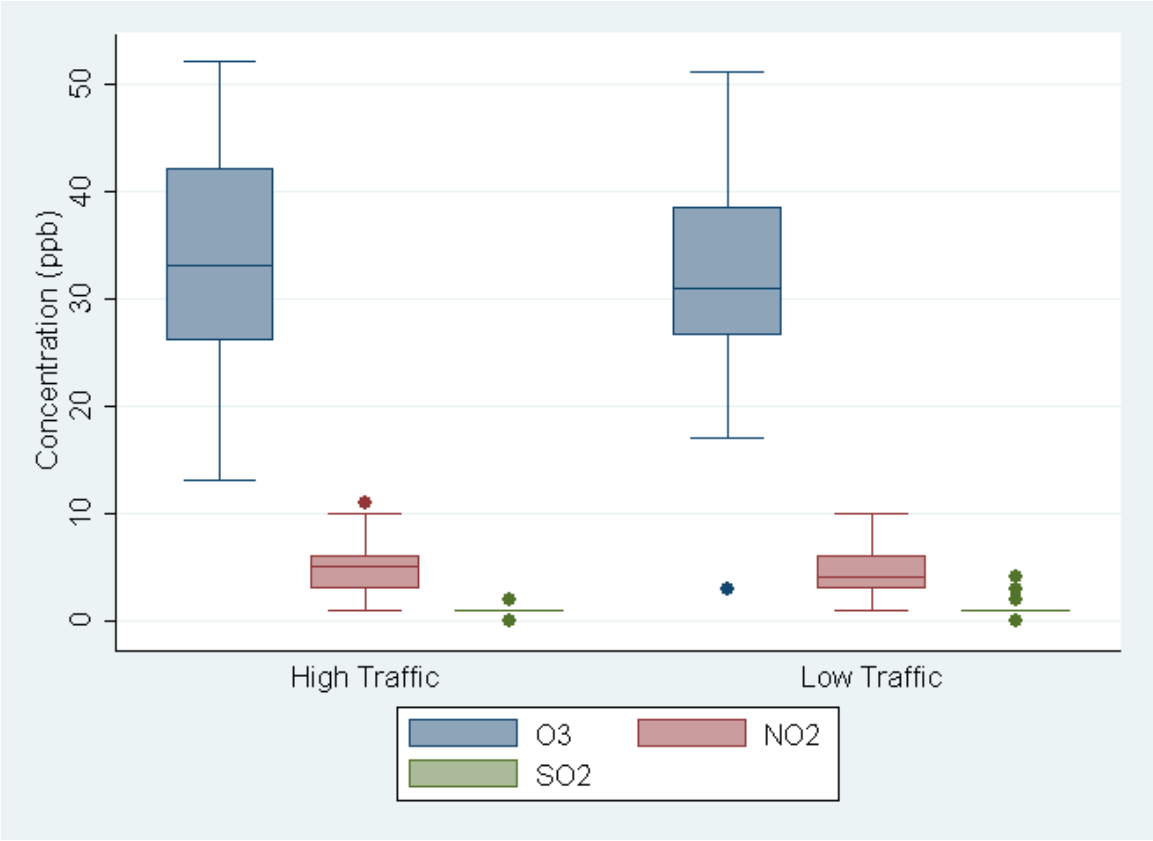

Supplement: (172 KB) PDF [file ehp.1003321.s001.pdf]
